# Long-term changes in dysnatremia incidence in the ICU: a shift from hyponatremia to hypernatremia

**DOI:** 10.1186/s13613-016-0124-x

**Published:** 2016-03-17

**Authors:** Annemieke Oude Lansink-Hartgring, Lara Hessels, Joachim Weigel, Anne Marie G. A. de Smet, Diederik Gommers, Prashant V. Nannan Panday, Ewout J. Hoorn, Maarten W. Nijsten

**Affiliations:** Department of Critical Care, University of Groningen, University Medical Center Groningen, Hanzeplein 1, 9700 RB Groningen, The Netherlands; Department of Intensive Care Adults, Erasmus MC, Rotterdam, The Netherlands; Department of Clinical Pharmacy and Pharmacology, University of Groningen, University Medical Center Groningen, Groningen, The Netherlands; Department of Internal Medicine, Erasmus MC, Rotterdam, The Netherlands

**Keywords:** Critical care, Sodium, Dysnatremia, Incidence, Hyponatremia, Hypernatremia, Infusion

## Abstract

**Background:**

Dysnatremia is associated with adverse outcome in critically ill patients. Changes in patients or treatment strategies may have affected the incidence of dysnatremia over time. We investigated long-term changes in the incidence of dysnatremia and analyzed its association with mortality.

**Methods:**

Over a 21-year period (1992–2012), all serum sodium measurements were analyzed retrospectively in two university hospital ICUs, up to day 28 of ICU admission for the presence of dysnatremia. The study period was divided into five periods. All serum sodium measurements were collected from the electronic databases of both ICUs. Serum sodium was measured at the clinical chemistry departments using standard methods. All sodium measurements were categorized in the following categories: <120, 120–124, 125–129, 130–134, 135–139, 140–145, 146–150, 151–155, 156–160, >160 mmol/L. Mortality was determined at 90 days after ICU admission.

**Results:**

In 80,571 ICU patients, 913,272 serum sodium measurements were analyzed. A striking shift in the pattern of ICU-acquired dysnatremias was observed: The incidence of hyponatremia almost halved (47–25 %, *p* < 0.001), whereas the incidence of hypernatremia nearly doubled (13–24 %, *p* < 0.001). Most hypernatremias developed after ICU admission, and the incidence of severe hypernatremia (sodium > 155 mmol/L) increased dramatically over the years. On ICU day 10 this incidence was 0.7 % in the 1992–1996 period, compared to 6.3 % in the 2009–2012 period (*p* < 0.001). More severe dysnatremia was associated with significantly higher mortality throughout the 21-year study period (*p* < 0.001).

**Conclusions:**

In two large Dutch cohorts, we observed a marked shift in the incidence of dysnatremia from hyponatremia to hypernatremia over two decades. As hypernatremia was mostly ICU acquired, this strongly suggests changes in treatment as underlying causes. This shift may be related to the increased use of sodium-containing infusions, diuretics, and hydrocortisone. As ICU-acquired hypernatremia is largely iatrogenic, it should be—to an important extent—preventable, and its incidence may be considered as an indicator of quality of care. Strategies to prevent hypernatremia deserve more emphasis; therefore, we recommend that further study should be focused on interventions to prevent the occurrence of dysnatremias during ICU stay.

**Electronic supplementary material:**

The online version of this article (doi:10.1186/s13613-016-0124-x) contains supplementary material, which is available to authorized users.

## Background

Deranged plasma sodium concentrations expose all cells to hypotonic or hypertonic stress. Clinical manifestations of dysnatremia are primarily neurological and rapid changes in plasma sodium in either direction can cause severe, permanent, and sometimes even lethal brain injury [1]. The reported prevalence of dysnatremia in the intensive care unit (ICU) ranges between 6.9 and 17.7 % and varies according to the time of onset (i.e., on admission or later during ICU stay), the threshold for diagnosis, and the population being assessed [2].

Patients in the ICU are at risk of developing both hyponatremia and hypernatremia. Critical illness may result in increased or reduced activity of the antidiuretic hormone [3, 4]. Additional factors that predispose to hypernatremia include a reduced urinary concentrating ability, the inability to express thirst, no free access to water, and increased insensible losses [5, 6]. In addition to critical illness per se, factors contributing to hyponatremia include excess use of hypotonic fluids and drugs stimulating antidiuretic hormone secretion [7].

The severity of hyponatremia on ICU admission is a demonstrated predictor of mortality [8]. Even slightly abnormal sodium levels on ICU admission are independently associated with poor outcome [2, 9]. Although ICU-acquired hyponatremia is less prevalent, it is also associated with an increased risk of hospital mortality [5]. ICU-acquired hypernatremia is also an independent risk factor for mortality and associated with increased ICU length of stay [10–13]. The relation between sodium derangement and mortality has been reported in medical, surgical, mixed, cardiac, cardiovascular surgery, trauma, and neurological ICUs [5, 10–15]. Finally, comparable to variability in serum glucose [16] or potassium [17], the magnitude of changes in sodium has also been associated with a higher risk of death in ICU patients [14, 15].

Based on our impression that hypernatremia has nowadays become more prevalent than hyponatremia in the ICU, we hypothesized that a shift in the incidence of hyponatremia and hypernatremia occurred during the past two decades. Therefore, the aim of this study was to analyze the long-term changes in the incidences of hyponatremia and hypernatremia in the ICU. Furthermore, we studied the association between dysnatremia and mortality.

## Patients and methods

This retrospective study was performed in two cohorts of adult ICU patients obtained from the two largest ICUs in The Netherlands, including the University Medical Center Groningen (44 bed unit) and the Erasmus Medical Center (48 bed unit). From the ICU of the University Medical Center in Groningen, all patients admitted between 1992 and 2011 were analyzed, and from the ICU of the Erasmus Medical Center in Rotterdam, all patients admitted between 1998 and 2012 were analyzed. The 21-year study period was divided into five periods to detect shift in time: 1992–1996, 1997–2000, 2001–2004, 2005–2008, and 2009–2012. Data on the type of admission (surgical, medical, etc.) were available for patients from Groningen but not from Rotterdam. Patients aged <15 years were excluded. Mortality was determined at 90 days after ICU admission. The anonymized data analysis in this study was performed in accordance with the guidelines and Dutch legislation, and it was approved by the medical ethical committees of our institutions (Medisch Ethische Commissie, UMC Groningen, METc 2014.264, MEC Erasmus MC MEC-2015-401). Since this concerned a retrospective study on routinely collected data, informed consent was not required by the ethical committees.

### Serum sodium measurements

All serum sodium measurements during ICU admission until day 28 were collected from the electronic databases of both ICUs. Serum sodium was measured at the clinical chemistry departments using standard methods (with a pre-analytic dilution, assuming a standard 7 % solid phase) or at the ICU with Radiometer 700 series blood gas analyzers with an ion-selective method that uses no pre-dilution. All sodium measurements (reference range 135–145 mmol/L) were categorized as follows: <120, 120–124, 125–129, 130–134, 135–139, 140–145, 146–150, 151–155, 156–160, >160 mmol/L. The so-called soccer field plots was generated for the 1992–1996 and the 2009–2012 periods to display the relation between ICU day and the relative incidence of dysnatremia in all patients between ICU day 1 and day 28. This way of presentation facilitates easier identification of trends in dysnatremia during the ICU stay. In these plots, dysnatremia was categorized into similar groups as defined earlier.

### Pharmacy data

The hospital pharmacy of the University Medical Center in Groningen provided a list of all infusions that were administered in the ICU over the period 1997 through 2011. In addition to the total volume infused, the mean sodium content was also calculated.

### Statistical analysis

Comparisons between means and medians were made with the Student’s *t* test and Mann–Whitney *U* test, respectively. Distributions were compared with the Chi-square test. Data are expressed as means with standard deviations. A *p* value <0.05 was considered statistically significant. Bonferroni correction was used where appropriate. Statistical analysis was performed with SPSS (IBM, version 22).

## Results

### Patient characteristics

In the two centers 80,571 consecutively admitted ICU patients were included, in whom a total of 913,272 serum sodium measurements were performed (55 % from Groningen). Sixty-four percentage of patients were male; mean age was 60 ± 16 years, with a mean of 11 ± 20 serum sodium measurements/patient. Table 1 shows the type of ICU admissions in Groningen over time. The case mix of patients remained relatively stable over the study period, except for a small increase in vascular and abdominal surgery, and a small decrease in cardiothoracic surgery. In the additional data file, the data selection (Additional file 1: Figure 1) and the frequency distribution of the number of admitted patients as a function of ICU day (Additional file 1: Figure 2) are provided.

**Table 1:** Type of ICU admissions in Groningen in the five time periods

	1992–1996	1997–2000	2001–2004	2005–2008	2009–2011	Total
	(*n* = 11,831) (%)	(*n* = 8,875) (%)	(*n* = 8,078) (%)	(*n* = 8,378) (%)	(*n* = 6857) (%)	(*n* = 44,019) (%)
Admission via emergency department	19	12	15	16	18	16
Vascular, abdominal and other surgery	14	17	20	21	21	18
Neurosurgery	11	10	12	14	13	12
Transplant	2	2	2	2	1	2
Cardiothoracic surgery	51	48	42	41	42	45
Trauma	4	4	5	5	5	4
Medical and miscellaneous	20	19	18	18	17	18

### Long-term changes in dysnatremia

**Fig. 1:**
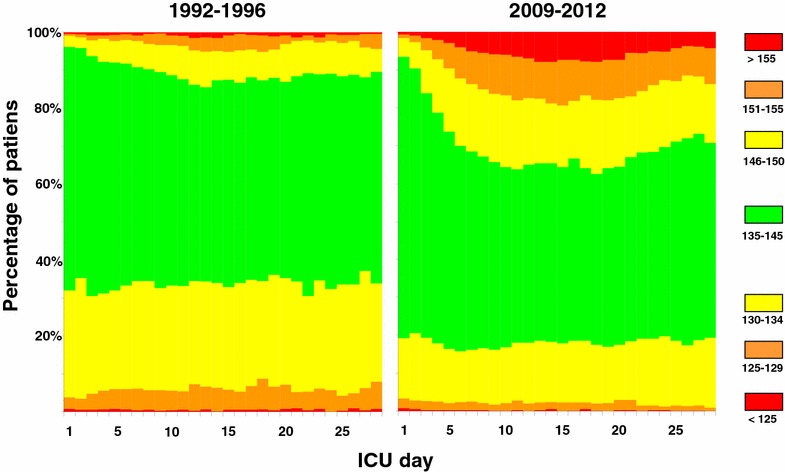
Time course of sodium derangements. Comparison of the development of hyponatremia and hypernatremia between the periods 1992–1996 and 2009–2012. Sodium levels within the reference range are shown in *green* and progressively more marked derangements in *yellow*, *orange*, and *red*, respectively

**Fig. 2:**
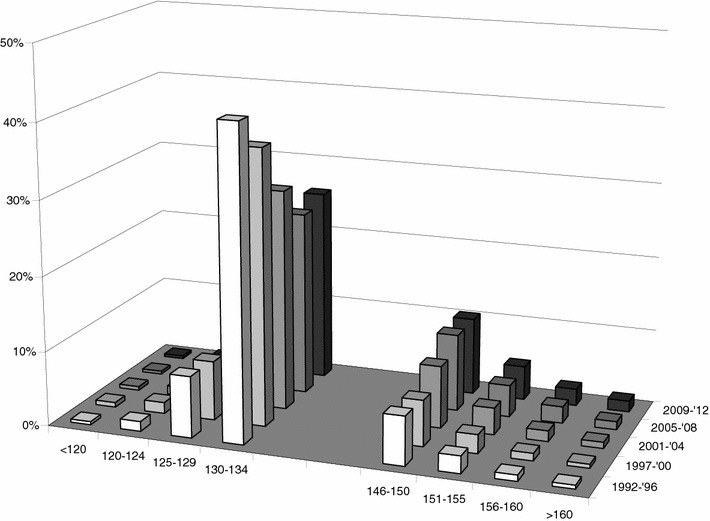
Incidence of dysnatremia in five time periods. For five time periods spanning 1992–2012 the incidence of various degrees of hyponatremia and hypernatremia is shown. Note that for clarity the two normonatremic categories are not shown

Figure 1 shows the change in distribution of serum sodium categories during ICU admission for the first time period (1992–1996, left panel) and the last time period (2009–2012, right panel). The figure clearly shows that hyponatremia was more common in the first period and that hypernatremia became more common in the last period. The figure also shows that in particular the incidence of hypernatremia increased during ICU admission (most notably in the first 2 weeks) and remained stable until day 20. On ICU day 10, for example, the incidence of hypernatremia >155 mmol/L rose from 0.7 % in 1992–1996 to 6.3 % in 2009–2012 (*p* < 0.001). Figure 2 shows a different graphical representation of the incidences of hyponatremia and hypernatremia during the five subsequent time periods. From this analysis, the decreasing prevalence of hyponatremia and the increasing incidence of hypernatremia are also clearly visible for the five consecutive time periods. For example, the incidence of hyponatremia <130 mmol/L decreased from 47 to 25 % (*p* < 0.001) from the first time period (1992–1996) to the last time period (2009–2012), whereas the incidence of hypernatremia > 150 mmol/L increased from 13 to 24 % (*p* < 0.001) in the same time periods. Over time the use of ion-selective sodium measurements has increased with the implementation of ICU-based point-of-care systems from 56 to 79 % (*p* < 0.001) in Groningen (Additional file 1: Table 1). However, the sodium levels determined by the ion-selective assay were 1.5 mmol/L (*p* < 0.001) lower in the Groningen ICU patients. As the ion-selective sodium levels were lower while being used more frequently, this should not have contributed to the observed trend toward higher sodium levels. Although a clear difference in albumin levels and in particular glucose levels was observed between 1992 and 1996 and 2009 and 2011, their statistical relation with sodium levels was very limited (Figures 5, 6 and regression analysis in Additional file 1).

**Fig. 3:**
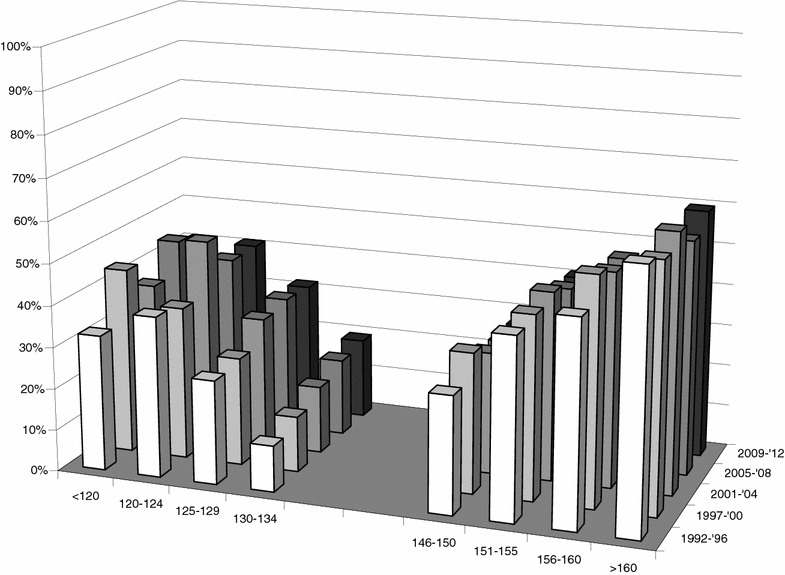
Dysnatremia and mortality in five time periods. Mortality at 90 days shows a U-shaped relation with sodium derangements. Note that the mortality associated with the various dysnatremia categories has not markedly changed over the years

When the changes in incidence of sodium abnormalities were analyzed separately for the Groningen and Rotterdam ICUs (Additional file 1: Table 3, Figure 3), both ICUs showed a trend toward hypernatremia. When analysis was performed for patients with hypernatremia >150 mmol/L all subgroups except transplantation showed a trend towards a higher incidence of hypernatremia in recent cohorts.

For the Groningen ICU, we also performed similar analyses for the top 35 routine laboratory measurements that were most frequently performed. With the exception of chloride, albumin, hemoglobin, and glucose, no important shifts over time were observed (Additional file 1: Figures 4, 5, 6). From the 1997–2000 to the 2009–2011 period, the mean sodium concentration of the infused fluids in the Groningen ICU increased from 100 to 107 mmol/L (*p* < 0.001; Additional file 1: Table 2).

### Mortality

Figure 3 shows the mortality rates with the various serum sodium categories and time periods. Dysnatremia was strongly associated with mortality and showed a U-shaped relationship. Figure 3 demonstrates that this relationship between dysnatremia and mortality remained largely unchanged over the 21-year study period. The overall mortality rose slightly from 13 % in 1992–1996 to 15 % in 1997–2000, 16 % in 2001–2004, 15 % in 2005–2008, and 16 % in 2009–2012 (*p* < 0.001).

## Discussion

In this large retrospective dual-center study, we observed a consistent and marked shift in the incidence of dysnatremia from hyponatremia to hypernatremia over a two-decade observation period. The incidence of hyponatremia nearly halved over the study period, whereas the incidence of hypernatremia almost doubled. The trend toward higher serum sodium levels was consistently observed in both centers and seemed to be more important in Groningen center. To our knowledge, this clear shift from hyponatremia to hypernatremia has not been reported before. The increased number of studies that address hypernatremia instead of hyponatremia may reflect increased awareness of this problem in other centers as well [5, 9, 10, 13–15]. Our observation that most hypernatremia typically developed after ICU admission strongly suggests that changes in therapy are involved in this trend.

Although Fig. 1 shows that hypernatremia at ICU admission has also increased over time, it is in particular the increase of hypernatremia after ICU admission that is striking. Although we do not have the data to evaluate the etiology of the dysnatremia, we do want to speculate on several factors which might have played a role. Two treatment-related factors that may have contributed to the shift from hyponatremia to hypernatremia are the less liberal use of intravenous fluids in combination with wider use of diuretic treatment and the increased use of steroids and in particular hydrocortisone. In 2006 the ARDS clinical network performed a study comparing a conservative strategy (mostly accomplished by administration of furosemide) with a liberal strategy of fluid management in patients with acute lung injury [18]. This trial provided evidence that more restrictive fluid management in critically ill patients results in improved lung function and shortened duration of mechanical ventilation and intensive care stay [19]. Fluid restriction may have contributed indirectly to the rising incidence of hypernatremia. Nevertheless, we have shown previously that more than one-third of the patients with ICU-acquired hypernatremia are actually still volume overloaded [11]. This phenomenon is explained by the combination of large volumes of (approximately) isotonic fluids and a reduced urinary concentrating ability. In line with these observations, it was recently shown that NaCl 0.9 % used to dilute drugs and keep catheters open contributes to the occurrence of ICU-acquired hypernatremia [20]. In the group of patients with ICU-acquired hypernatremia, the plasma creatinine and dose of furosemide were also higher, again suggesting compromised urinary concentrating ability as an important contributor to hypernatremia.

The role of hydrocortisone in the treatment of septic shock has evolved over the last two decades. In 2002 a French multicenter RCT of patients in vasopressor-unresponsive septic shock showed significant shock reversal and reduction of mortality rate in patients with relative adrenal insufficiency [21]. This led to a more prominent role of hydrocortisone in guidelines for patients with septic shock. In 2008 the CORTICUS trial, a large European multicenter study failed to show a mortality benefit with steroid therapy [22]. Nevertheless, a recent study in a university ICU in The Netherlands showed that hydrocortisone was the seventh most frequently administered drug [20]. Even after several systematic analyses, the role of hydrocortisone in septic shock is still not settled [23, 24], and a systematic review identified a greater risk of hypernatremia with the use of corticosteroids [23]. We have no data on renal function and the use of renal replacement therapy (RRT) over this period, but RRT did not change in this respect, namely continuous veno-venous hemofiltration with a substitution fluid with a sodium content of 140 mmol/L. The widespread use of dopamine in past decades may have helped to avoid hypernatremia, since dopamine is a natriuretic agent.

The clinical implication of our observations and those of others is that early identification of hypernatremia or preferably impending hypernatremia may help to reduce the incidence, severity, and duration of hypernatremia. It has been proposed to consider the development of hypernatremia during ICU stay as an indicator of quality of care, because ICU patients depend fully on the competence of the medical staff for prescribing fluids and these patients are frequently monitored with sampling of blood [25]. The impact from dysnatremia on morbidity and mortality leads to extensive burden on healthcare resources [26]. It is sobering to note that this proposal to prevent hypernatremia [25] was made more than 15 years ago, and yet its incidence has only increased. Nevertheless, we still believe it may be possible to achieve the best of both worlds, i.e., combine the low hypernatremia levels observed in the 1990s and the low hyponatremia levels observed today. Since hypernatremia is a condition that takes several days to develop and also takes a relatively long time to correct (Fig. 1), prevention would be the most desirable strategy. Our own data (Additional file 1: Table 1) show that although the total infused volume decreased, unfortunately the sodium concentration of infused fluids has only increased over the years. A strategy of timely administration of infusion fluids with lower sodium content in the face of imminent hypernatremia seems reasonable to pursue. In this regard, the trend to use balanced fluids with lower sodium concentrations than the 154 mmol/L in NaCl 0.9 % may help [27, 28]. Since patients more often arrive at the ICU with hypernatremia, this is also relevant for the emergency department and operating room. When other risk factors for hypernatremia such as the administration of furosemide [20] or hydrocortisone are present, an even earlier switch to infusions with minimal sodium concentrations may be desirable. Monitoring of sodium concentrations is facilitated with modern point-of-care equipment. In the slipstream of glucose control, we have demonstrated that careful computer-guided potassium control is feasible with a clear reduction of abnormal potassium levels [17]. In fact, integration of potassium regulation with glucose control was very effective with marginal extra costs or time spent [29]. But implementing computerized sodium control will be considerably more challenging since more variables have to be taken into account. Although no study has shown that treatment of dysnatremia reduces mortality, a large multicenter observational study recently showed that successful rapid correction of dysnatremia was independently associated with survival [30]. The 28-day mortality in patients whose dysnatremia was corrected within 48 h was not significantly different from that in patients with normal serum sodium concentrations on ICU admission [30]. This finding suggests that the association between dysnatremia and mortality may be causal and could be improved by timely correction, although conclusive evidence should come from a randomized trial.

This study has a number of limitations. First, the identified trends in our two ICUs (large ICUs in university hospitals in The Netherlands) may not apply to other types of ICUs. Second, in addition to treatment-related factors, patient-related factors may also have played a role. For example, admissions for traumatic brain injury may have changed over the years, but this was not clearly reflected in our analysis of type of ICU admissions (Table 1). Also hypernatremia in patients with severe traumatic brain injury typically develops within the first three days [31] and not at the considerably later time as shown in Fig. 1. We do not have the data on shifts in medication usage in our two centers to support out speculation on the etiology of the dysnatremia. Data on sodium concentration in mmol/L of the administered infusions in the Groningen ICU show a significant increase over time (Additional file 1: Table 1). Data on hyperosmolar dye contrast administration, the use of citrate anticoagulation on RRT, or (par)enteral nutrition were also not analyzed. We were also not able to identify complications of dysnatremia. Likewise we could not reliably determine how often a disastrous complication such as central pontine myelinolysis occurred [32]. Finally, historical comparisons are vulnerable to all kinds of system changes that inevitably happen over time. This may be the case for sodium measurement. One study showed that the discrepancy between the direct assay and the indirect assay (which includes a dilution step) became larger as plasma albumin decreased [33], [34]. Because patients in the ICU are often hypoalbuminemic, this may predispose to pseudohypernatremia. However, the point-of-care measurements that use a direct sodium assay became more prevalent during the study period. Thus, pseudohypernatremia may have been more common in the past, making the incidence of true hypernatremia in earlier time periods even lower.

## Conclusions

In two large cohorts of ICU patients, we found a shift in the incidence of dysnatremias. The incidence of hyponatremia decreased over the study period, whereas the incidence of hypernatremia increased. We suggest this shift is related to the increased use of diuretics and hydrocortisone. As ICU-acquired hypernatremia is often iatrogenic, it thus may be—to an important extent—preventable, and its incidence may be considered as a quality indicator. The relation of dysnatremia with mortality remained unchanged over the 21-year study period; therefore, we recommend that further study should be focused on interventions to prevent the occurrence of dysnatremias during ICU stay.

## Additional file

10.1186/s13613-016-0124-x Changes in (relative) use of ion-selective sodium measurements in the Groningen ICU and the associated sodium concentrations measured. The use of ion-selective sodium measurements has increased with the implementation of ICU-based point-of-care systems from 56% to 79% (P < 0.001) in Groningen. **Table S2.** Bulk infusion fluid use. All infusions administered in the Groningen ICU per epoch. When the change in mean sodium concentration of all infusions was determined, a rise of 7% was observed (P < 0.001).** Tables S3a and b.** Distribution of epoch-dependent sodium abnormalities for the two ICU’s.** Table S4.** Type of ICU admissions and incidence of hypernatremia >150 mmol/L. For all categories except transplantation. A clear increase (P < 0.001) in the incidence of hypernatremia is observed.** Figure S1.** Flowchart patient and data selection.** Figure S2.** Fraction of patients over time. This study examined sodium levels up to 28 days after ICU admission.** Figure S3.** Incidence of dysnatremia in five time periods for Groningen only. Note that for clarity the two normonatremic categories are not shown.** Figure S4a and b.** Soccerfield plots of chloride abnormalitiesin 1992–’96 and 2009–’11.** Figure S5a and b.** Soccerfield plots of albumin abnormalities in 1992–’96 and 2009–’11.** Figure S6a and b.** Soccerfield plots of glucose abnormalities in 1992–’96 and 2009–’11.

## Abbreviations

ICU: intensive care unit
RRT: renal replacement therapy
