# Supplementary material for: Long-term changes in dysnatremia incidence in the ICU: a shift from hyponatremia to hypernatremia
Source: Ann Intensive Care. 2016 Mar 17;6:22. doi: 10.1186/s13613-016-0124-x (PMC4794471; doi:10.1186/s13613-016-0124-x)
Supplement: Supplementary file 1 — 10.1186/s13613-016-0124-x Changes in (relative) use of ion-selective sodium measurements in the Groningen ICU and the associated sodium concentrations measured. The use of ion-selective sodium measurements has increased with the implementation of ICU-based point-of-care systems from 56% to 79% (P < 0.001) in Groningen. Table S2. Bulk infusion fluid use. All infusions administered in the Groningen ICU per epoch. When the change in mean sodium concentration of all infusions was determined, a rise of 7% was observed (P < 0.001). Tables S3a and b. Distribution of epoch-dependent sodium abnormalities for the two ICU’s. Table S4. Type of ICU admissions and incidence of hypernatremia >150 mmol/L. For all categories except transplantation. A clear increase (P < 0.001) in the incidence of hypernatremia is observed. Figure S1. Flowchart patient and data selection. Figure S2. Fraction of patients over time. This study examined sodium levels up to 28 days after ICU admission. Figure S3. Incidence of dysnatremia in five time periods for Groningen only. Note that for clarity the two normonatremic categories are not shown. Figure S4a and b. Soccerfield plots of chloride abnormalitiesin 1992–’96 and 2009–’11. Figure S5a and b. Soccerfield plots of albumin abnormalities in 1992–’96 and 2009–’11. Figure S6a and b. Soccerfield plots of glucose abnormalities in 1992–’96 and 2009–’11. [file 13613_2016_124_MOESM1_ESM.pdf]

Additional file for
"Long term changes in dysnatremia incidence in the ICU: A shift from hyponatremia to hypernatremia"
by Annemieke Oude Lansink et al.

Note: the main manuscript contains **Table 1** and **Figure 1, Figure 2 and Figure 3**.

This additional file contains:

Page 2

Table 2:
Changes in (relative) use of ion-selective sodium measurements in the Groningen ICU and the associated sodium concentrations measured.

Table 3:
Administered infusions and their sodium content over time in the Groningen ICU.

Page 3

Tables 4a and 4b:
Incidence of sodium abnormalities over time for the Groningen ICU and the Rotterdam ICU.

Page 4

Table 5:
Type of ICU admissions and incidence of hypernatremia over time**.**

Page 5

Figure 4:
Flowchart of patient and data selection.

Page 6

Figure 5:
Fraction of patients compared to number admitted over time in the Groningen ICU.

Page 7

Figure 6:
Incidence of dysnatremia in five time periods for Groningen ICU.

Page 8

Figure 7a and 7b:
Soccerfield plots of chloride abnormalities in 1992-'96 and 2009-'11

Page 9

Figure 8a and 8b:
Soccerfield plots of albumin abnormalities in 1992-'96 and 2009-'11

Page 10

Figure 9a and 9b:
Soccerfield plots of glucose abnormalities in 1992-'96 and 2009-'11

Page 11

Regression analysis of sodium as dependent variable.

Table 2. Changes in (relative) use of ion-selective sodium measurements in the Groningen ICU and the associated sodium concentrations measured.

Epoch	Number	Percentage of assays that is ion-selective	Mean ±SD [Na^+] with conventional assay mmol/L	Mean ±SD [Na^+] with ion-selective assay mmol/L
1992-'96	85918	56%	138.3 ±6.0	136.6 ±4.0
1997-'00	66848	54%	138.1 ±6.1	136.2 ±4.3
2001-'04	71570	52%	140.3 ±6.9	136.9 ±4.7
2005-'08	136304	67%	141.6 ±6.2	139.6 ±5.5
2009-'12	172506	79%	142.3 ±6.1	139.9 ±5.8
Total	533146	66%	140.2 ±6.5	138.7 ±5.5

Legend for Table 2.
Over time the use of ion-selective sodium measurements has increased with the implementation of ICU-based point-of-care systems from 56% to 79% (P<0.001) in Groningen. The sodium levels reported by the ion-selective assay are 1.5 mmol/L (P<0.001) lower in the Groningen ICU patients. The sodiums of the Rotterdam ICU were not split, since this ICU has used conventional assays throughout the study period. As the ion-selective sodium levels were lower whilst being used more frequently, this should not have contributed to the observed trend towards higher sodium levels.

Table 3. Bulk infusion fluid use.

Epoch	Sodium (mole)	Volume (L)	Concentration (mmol/L)
**1992-1996**	NA	NA	NA.
**1997-2000**	11,895	119,152	99.8
**2001-2004**	12,571	119,443	105.2
**2005-2008**	13,676	127,908	106.9
**2009-2011**	10,987	102,837	106.8

Legend for Table 3.
All infusions administered in the Groningen ICU per epoch, obtained from hospital pharmacy data. When the change in mean sodium concentration of all infusions was determined, a rise of 7% was observed (P < 0.001). NA: not available

Table 4a. Incidence of sodium abnormalities over time in Groningen ICU.

	<120	120-124	125-129	130-134	146-150	151-155	156-160	>160	N
**1992-'96**	0.4%	1.3%	8.2%	41.7%	6.6%	2.3%	0.8%	0.5%	10925
**1997-'00**	0.4%	1.4%	8.8%	43.4%	5.3%	2.2%	0.9%	0.5%	8679
**2001-'04**	0.3%	1.0%	6.2%	34.9%	7.6%	3.4%	1.6%	1.2%	8152
**2005-'08**	0.2%	0.6%	3.8%	24.5%	11.3%	4.8%	2.4%	1.2%	8832
**2009-'12**	0.3%	0.8%	4.2%	27.3%	12.4%	5.4%	2.8%	1.2%	7535

Table 4b. Incidence of sodium abnormalities over time in Rotterdam ICU.

	<120	120-124	125-129	130-134	146-150	151-155	156-160	>160	N
**1992-'96**	NA	NA	NA	NA	NA	NA	NA	NA	
**1997-'00**	1.0%	1.7%	6.3%	24.7%	7.8%	3.1%	1.2%	0.5%	4645
**2001-'04**	0.7%	1.3%	6.2%	24.5%	9.0%	3.8%	1.3%	0.5%	8434
**2005-'08**	0.5%	1.1%	5.3%	24.8%	9.6%	3.8%	2.1%	1.0%	11197
**2009-'12**	0.5%	1.0%	4.9%	24.8%	9.2%	4.1%	1.9%	1.6%	12326

Legend for Tables 4a and 4b.
Distribution of epoch-dependent sodium-abnormalities for the two ICU's. For each patient the most extreme sodium abnormality in either direction was recorded. N is number of patients per epoch; NA: not available.

Table 5: Type of ICU admissions and incidence of hypernatremia >150 mmol/L.

Absolute numbers

	1992-'96	1997-'00	2001-'04	2005-'08	2009-'11
**Medical**	109	52	111	120	106
**Vascular, abdominal, miscellaneous**	68	65	102	185	153
**Neurosurgery**	53	30	55	105	71
**Transplantation**	42	17	13	25	16
**Cardiothoracic surgery**	64	70	65	127	163
**Trauma**	23	20	49	70	76

Percentages

	1992-'96	1997-'00	2001-'04	2005-'08	2009-'11
**Medical**	8.7%	8.2%	17.5%	18.2%	21.7%
**Vascular. abdominal. miscellaneous**	4.4%	4.4%	6.5%	10.1%	9.5%
**Neurosurgery**	4.6%	3.3%	5.6%	8.5%	7.2%
**Transplantation**	23.1%	10.0%	8.1%	12.7%	12.6%
**Cardiothoracic surgery**	1.1%	1.7%	1.9%	3.5%	5.2%
**Trauma**	5.5%	5.5%	12.6%	17.8%	21.1%
**Miscellaneous**	5.5%	6.3%	10.9%	12.2%	15.3%

Legend for table 5.
Numbers of ICU patients with hypernatremia over 150 mmol/L at any time point during ICU stay in Groningen ICU. For all categories except transplantation. a clear increase ($P<0.001$) in the incidence of hypernatremia is observed.

Figure 4: Flowchart patient and data selection.

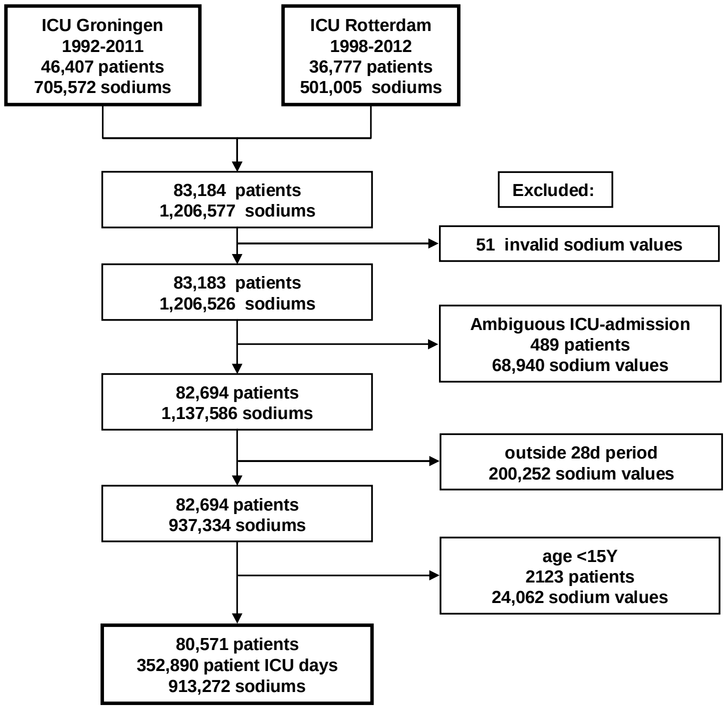

Legend to figure 4.
Depiction of the various data reduction steps that resulted in the selection of the 80.571 patients from the Groningen ICU and Rotterdam ICU. Invalid values. values from children. values obtained outside the ICU and data from later than 28 days were excluded.

Figure 5: Fraction of patients over time.

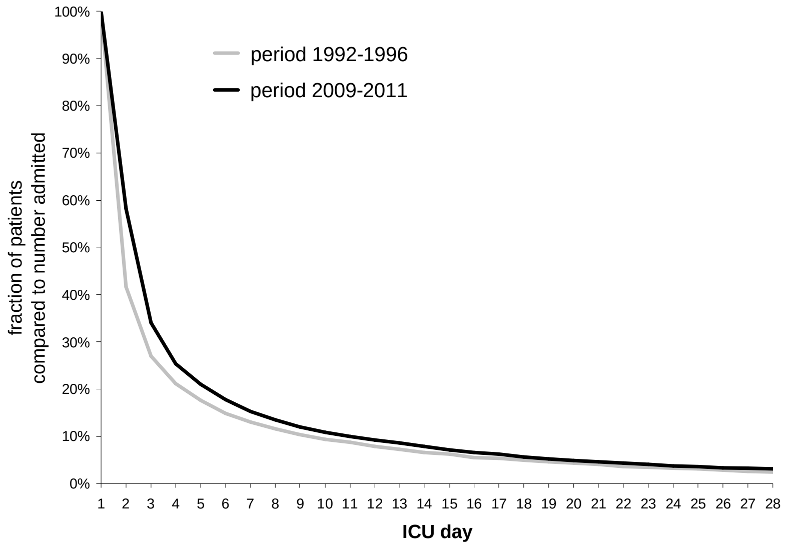

Legend to figure 5.
This study examined sodium levels up to 28 days after ICU admission. As a reference. these curves show the number of patients that are still admitted at the Groningen ICU over time.

Figure 6.
Incidence of dysnatremia in five time periods for Groningen only.

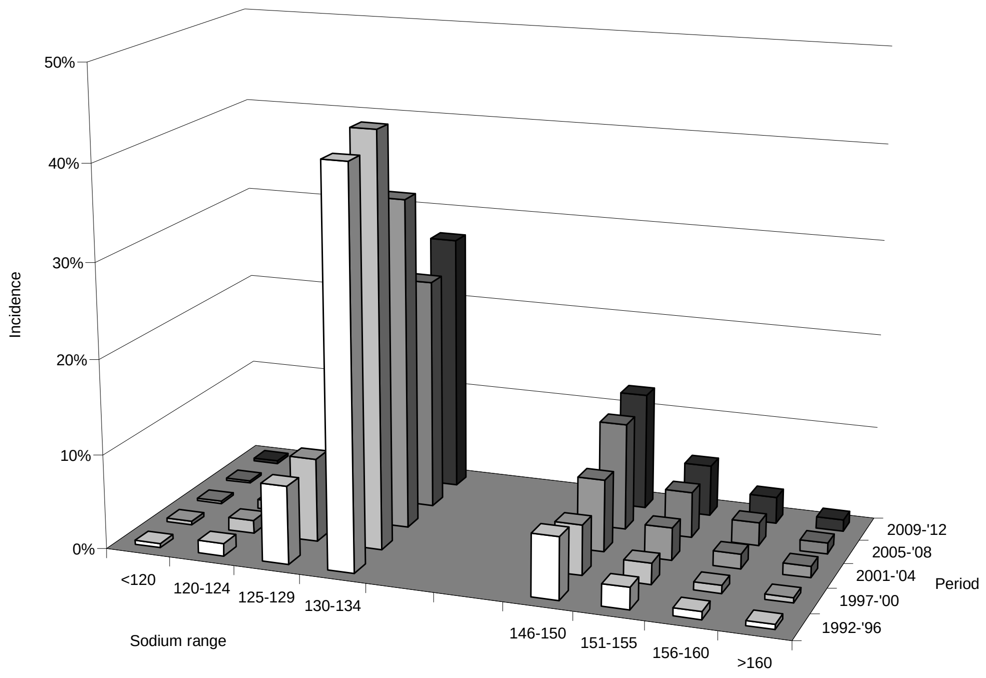

Legend to Figure 6: For five time periods spanning 1992 to 2012 the incidence of various degrees of hyponatremia and hypernatremia are shown for the Groningen ICU only. When compared with Figure2 that shows the combined incidence of the Groningen and Rotterdam ICU's the same pattern of an increase of hypernatremia and in particular of marked hypernatremia. Note that for clarity the two normonatremic categories are not shown. Underlying data are shown in table

Figure 7a and 7b: Soccerfield plots of chloride abnormalities in 1992-'96 and 2009-'11

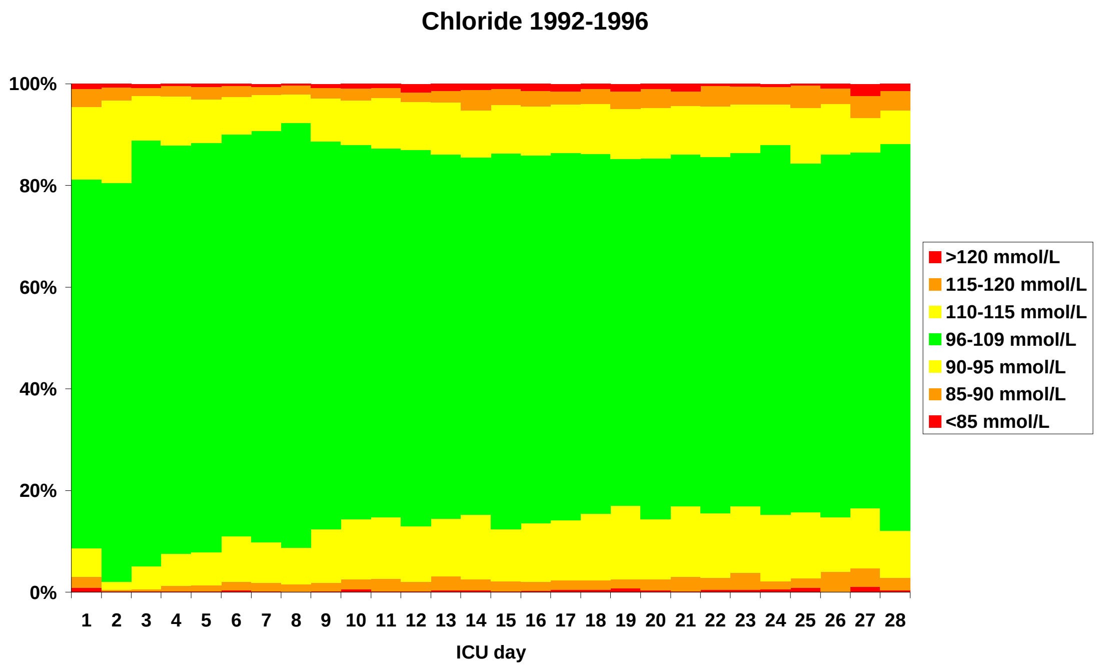

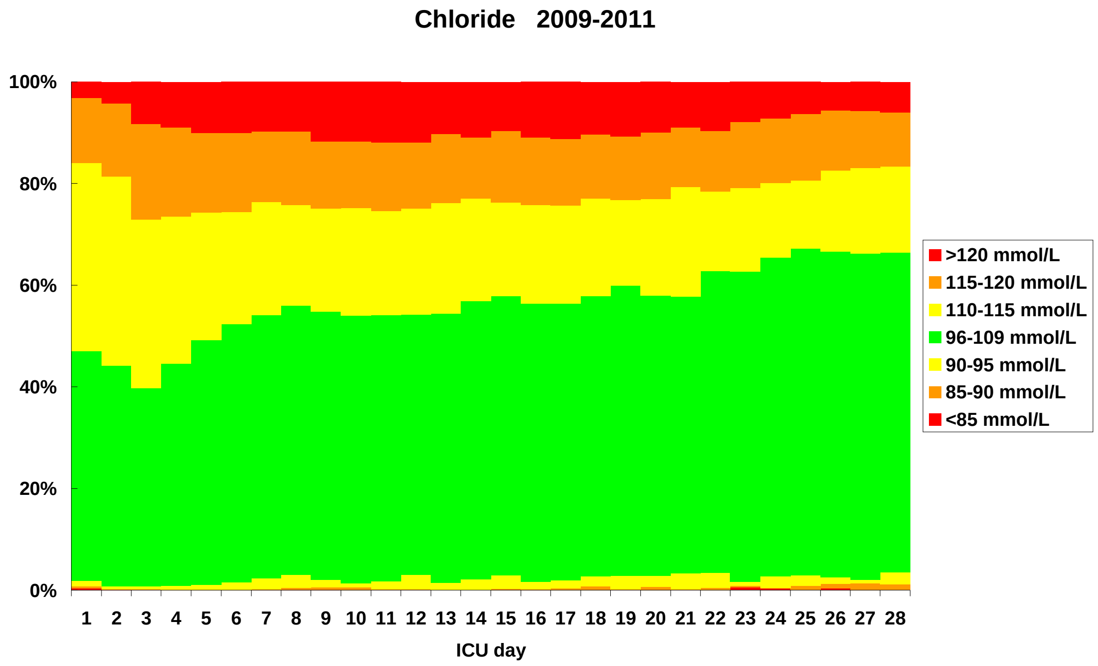

Legend to figures 7a and 7b. For each day in the Groningen ICU for each patient the median chloride value was determined. The distribution of all these values is color-coded with the reference range in green. Note that during the later epoch higher chloride levels develop after ICU-admission. Note the strong similarity with the same type of plot for sodium. suggesting NaCl administration as a common cause.

Figure 8a and 8b: Soccerfield plots of albumin abnormalities in 1992-'96 and 2009-'11

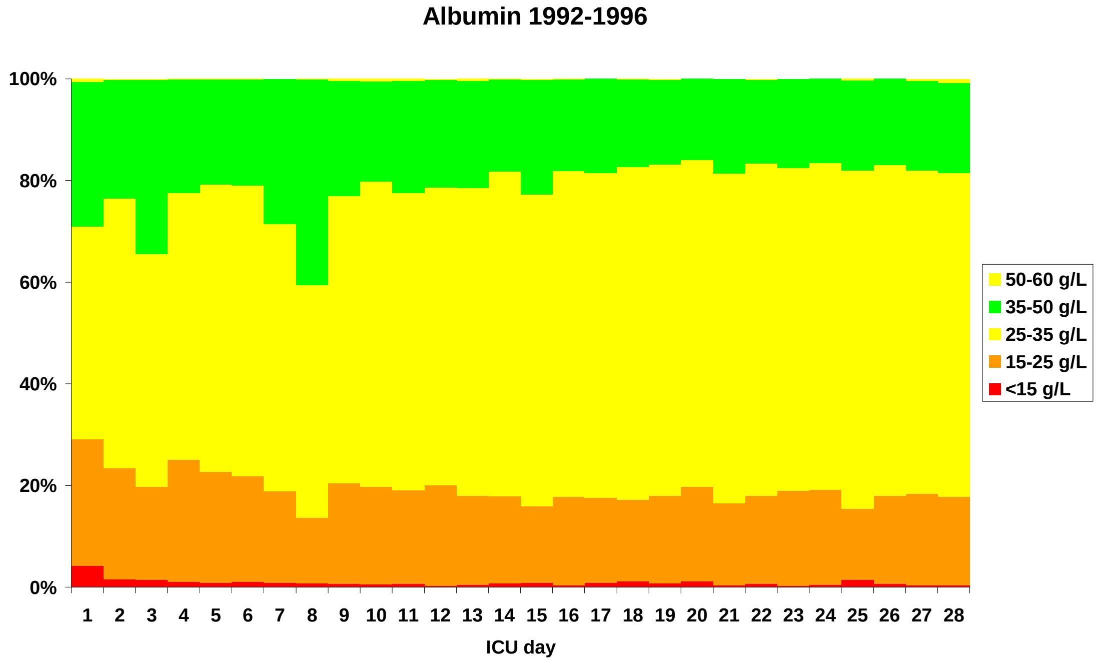

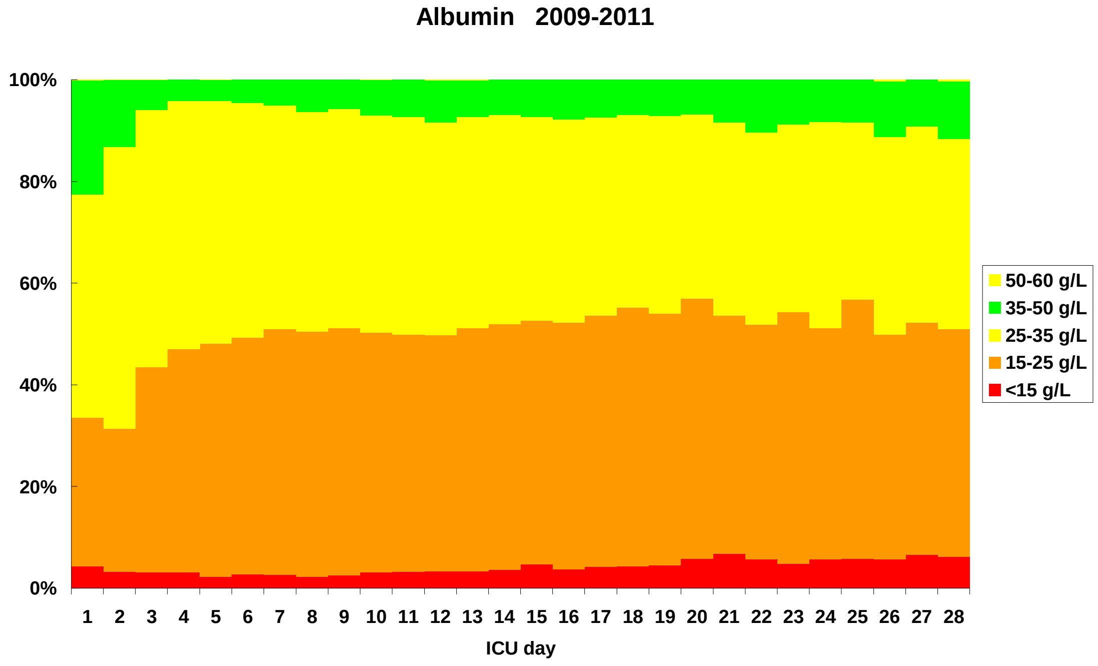

Legend to figures 8a and b. For each ICU in the Groningen ICU for each patient the median albumin value was determined. The distribution of all these values is color-coded with the reference range in green. Note how in the more recent epoch much more deranged albumin levels are observed (and accepted).

Figure 9a and 9b: Soccerfield plots of glucose abnormalities in 1992-'96 and 2009-'11

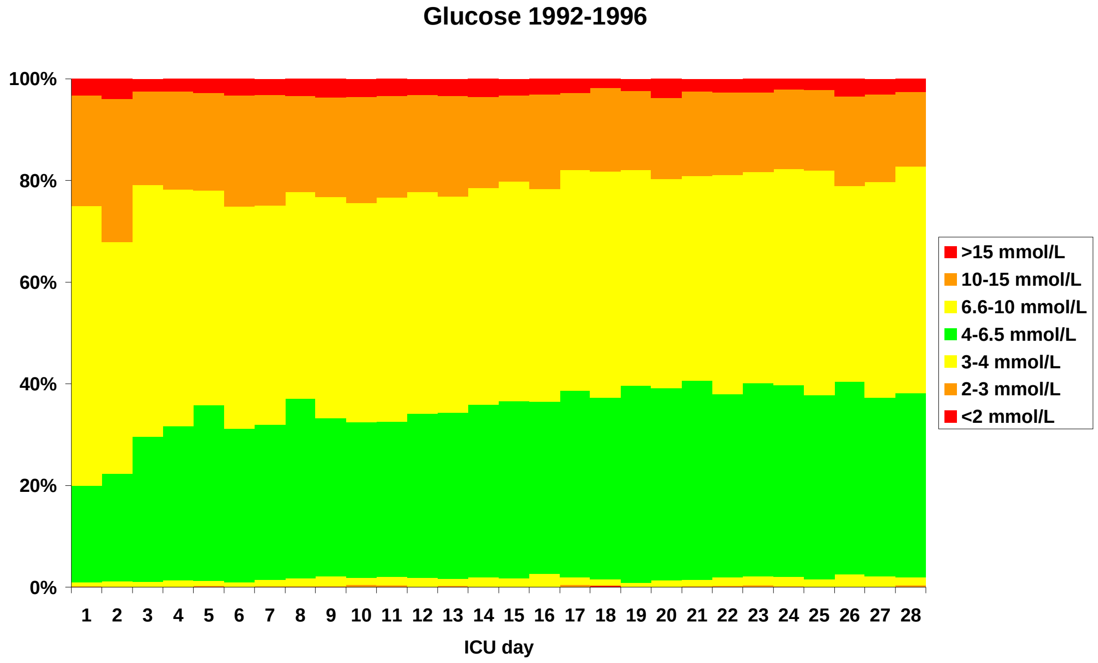

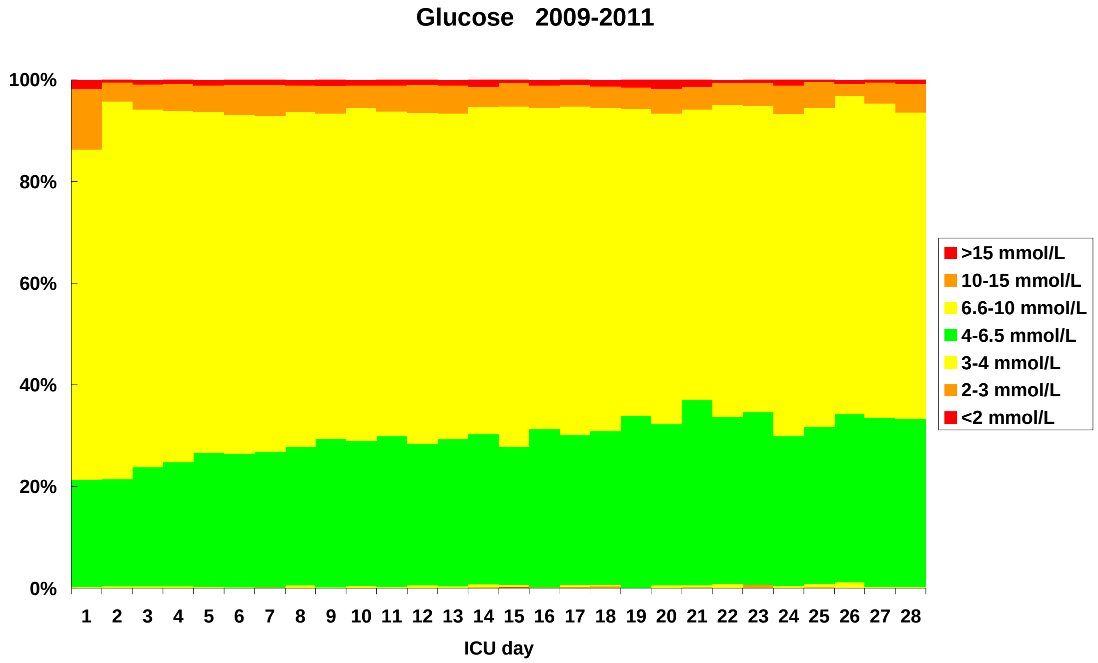

Legend to figures 9a and 9b. Example of medical policy-related change in laboratory value over time. For each day in the Groningen ICU for each patient the median glucose value was determined. The distribution of all these values is color-coded with the reference range in green. Note that during the later epoch glucose levels are better regulated. not as a result of changes in case mix. but as a result of active glucose control.

Linear regression model with median daily sodium as dependent variable for all ICU days.

	B (95%CI)	P
**constant**	138	
**ICU-day**	0,31 (0.30 to 0.33)	<0.0001
**(ICU-day)^2**	-0,011 (-0.12 to -0.10)	<0.0001
**Albumin**	-0.031 (-0.34 to -0.27)	<0.0001
**Glucose**	-0.054 (-0.065 to -0.043)	<0.0001

Linear regression model with median daily sodium as dependent variable

Legend. For 149,000 ICU days albumin, sodium and glucose levels were available for the Groningen ICU. This model includes ICU-day.
A mild inverse relation between glucose (in mmol/L) and sodium was observed. Thus a 10 mmol/L increase in glucose (180 mg/dL) is associated with a decrease of 0.5 mmol/L in sodium.
Likewise a 10 g/L decrease in albumin was associated with a 0.3 mmol/L rise in sodium.

Linear regression model with median daily sodium as dependent variable on ICU day 1.

	B (95%CI)	P
**constant**	137	
**Albumin**	0.019 (0.011 to 0.027)	0.001
**Glucose**	-0.005 (-0.025 to +0.016)	0.67

Linear regression model with median daily sodium as dependent variable

Legend. For 17,039 patients sodium, glucose and albumin were available on the date of ICU-admission. Only albumin has a slight relation with sodium levels.
